# Molecular and serological investigations of Batai virus in cattle and goats in the border area of Yunnan, China (2021–2022)

**DOI:** 10.3389/fvets.2024.1433699

**Published:** 2024-07-31

**Authors:** Zishuo Lu, Xingxiu Yan, Guiying Fan, Lixia Li, Xiutao Sun, Huijun Lu, Ningyi Jin, Hao Liu, Wenchao Sun

**Affiliations:** ^1^School of Life Sciences and Engineering, Foshan University, Foshan, China; ^2^Honghe Animal Disease Prevention and Control Center, Mengzi, China; ^3^Institute of Military Veterinary, Academy of Military Medical Sciences, Changchun, China; ^4^Wenzhou Key Laboratory for Virology and Immunology, Institute of Virology, Wenzhou University, Wenzhou, China

**Keywords:** Batai virus, serological survey, protein expression, ELISA, livestock

## Abstract

**Introduction:**

Batai virus (BATV), a zoonotic pathogen transmitted by mosquitoes, infects vertebrates, including livestock, birds, and humans. Although BATV has been detected and isolated in mosquitoes in Yunnan Province, China, there have been no reports of livestock infection. Thus, we conducted a molecular and serological investigation of BATV in cattle and goat sera collected in spring and autumn from 2021 to 2022 in Honghe Prefecture, Yunnan Province, on the China-Vietnam border.

**Methods:**

We used indirect enzyme-linked immunosorbent assays and reverse transcription real-time PCR (RT-qPCR) to test 929 cattle and 973 goat serum samples.

**Results:**

BATV antibodies were detected in 262/929 (28.2%) cattle and 263/973 (27.0%) goat serum samples. RT-qPCR did not detect BATV RNA.

**Discussion:**

The positive rate of BATV serum antibodies in cattle and goats in Luxi County was higher compared with other areas, and it was also higher in autumn compared with spring, which may be related to climate, temperature, and mosquito density. Although our findings indicated the presence of BATV infection in livestock in the region, RT-qPCR did not detect BATV RNA. Therefore, BATV monitoring in cattle and goats should be heightened in autumn, and the scope of host monitoring should be expanded to clarify the hosts and vectors of BATV infection.

## 1 Introduction

Batai virus (BATV) (family *Peribunyaviridae*, genus *Orthobunyavirus*, species *Orthobunyavirus bataiense*) is an enveloped arbovirus with a diameter of approximately 80 nm. BATV infects livestock, poultry, birds, and wild animals, making it one of the most widely distributed members of the *Orthobunyavirus* genus (1–3). *Bunyaviruses* have a genome with three unique species of single-stranded negative sense RNA: L, M, and S. The S segment codes for N and NSs, the M segment codes for Gc, Gn, and NSm, and the Gc-protein is highly immunogenic and a major target for neutralizing antibodies (4). The L segment encodes the large virion protein (L) responsible for genome transcription and replication (5). This genus comprises segmented RNA viruses with high homology between segments, facilitating easy genetic recombination (6). For example, the small (S) and large (L) segments of Bunyamwera virus and the medium (M) segment of BATV recombined to form a new virus strain, named Ngari virus, which has caused two large-scale human hemorrhagic fever outbreaks in Africa (7, 8). In nature, BATV spreads through a mosquito-vertebrate-mosquito pathway, with *Culex*, *Anopheles*, and *Aedes* as its main vectors. BATV can infect vertebrates, including livestock, birds, and humans (3). In humans, it can cause fever and aseptic meningitis. Infected livestock may show flu-like symptoms, severe neurological issues, and abortion (2, 7). BATV was first isolated in 1955 from *Culex* mosquitoes in Kuala Lumpur, Malaysia, and has since appeared and spread in many parts of Asia, Europe, and Africa (1). To date, BATV-neutralizing antibodies have been detected in poultry, cattle, goats, sheep, dogs, camels, reindeer, and rodents (9–13). In 1998, BATV strain YN92-4 was isolated from *Anopheles philippinensis* in the lower reaches of the Lancang River in Yunnan Province, China, and BATV-neutralizing antibodies were detected in the serum of 4.2% (5/120) of patients with febrile disease, confirming BATV prevalence in Yunnan Province (14). In 2012, the BATV strain NM-12 was isolated from febrile cattle serum in Inner Mongolia (15), and in 2014, the duck-origin ZJ2014 strain was isolated from Muscovy duck (*Cairina moschate*) tissue samples in Zhejiang (16). BATV is likely prevalent in China and its host range is increasing, but there is limited research on its diagnostic methods and epidemiology. The province with the highest prevalence of arboviruses in China is Yunnan, which neighbors Laos, Myanmar, and Vietnam, where 13 types have been identified (17). Very few serological investigations of BATV in livestock have been undertaken. Thus, conducting a molecular and serological investigation of BATV in this region to clarify the positive rate and prevalence of BATV antibodies and BATV RNA in livestock serum is of great importance to public health and safety and will contribute to the prevention and control of arbovirus infection.

## 2 Materials and methods

### 2.1 Animal serum collection

From 2021 to 2022, a total of 929 cattle and 973 goat serum samples were collected from Jianshui County (23°38′5.96″N,102°49′37.16″E), Mile City (24°24′38.12″N,103°24′53.96″E), and Luxi County (24°31′55.56″ N,103°45′58.43″E) in Honghe Prefecture, Yunnan Province. Samples were collected from various populations in three regions, with 11 cattle farms in one region, 28 in another, and 17 in the third. The respective number of goat farms in each region is 23, 24, and 13. The blood samples were collected from healthy cattle and goats, centrifuged to obtain serum, classified by region, type, and collection date, and refrigerated for future analysis after aliquoting.

### 2.2 Cells and viruses

Mouse neuroblastoma N2a cells that were preserved in our laboratory were maintained in Dulbecco modified Eagle medium (DMEM) supplemented with 10% fetal bovine serum (FBS) and 1% penicillin–streptomycin (PS, 100×) at 37°C under 5% CO_2_. Following BATV infection, the cells were cultured in PS-DMEM with 2% FBS. The NM/12 BATV strain was used for virus neutralization test.

### 2.3 Expression and purification of BATV-Gc_189aa–239aa_ protein

We investigated the serum prevalence of BATV infection in the cattle and goat serum samples using indirect ELISA. The sequence of the M segment of BATV NM/12 was downloaded from the National Center for Biotechnology Information^1^ database, and the protein antigenicity was analyzed using the Immune Epitope Database.^2^ The amino acid sequence at positions 189–239 of Gc protein was amplified and inserted at the *Eco*RI-*Xho*I cleavage site of the prokaryotic expression vector pET-32a(+), subsequently named pET32a-BATV-Gc_189aa − 239aa_. The recombinant plasmid was transformed into *Escherichia coli* BL21-competent cells to obtain cloned bacteria. Protein expression was induced at 37°C for 10 h with isopropyl β-d-1-thiogalactopyranoside (IPTG) at a final concentration of 0.6 mM. The recombinant protein was extracted and purified using BeyoGold His-tag Purification Resin (BeyoGold, China), and its identity was confirmed using western blotting and sodium dodecyl sulfate-polyacrylamide gel electrophoresis (SDS-PAGE). Coomassie bright blue staining followed SDS-PAGE electrophoresis. The purified protein was transferred to a PVDF membrane using SDS-PAGE electrophoresis and washed three times. It was then incubated with a His-Tag(2A8) Mouse mAb (Abmart, China) at 4°C overnight. After washing three times, an HRP-conjugated Affinipure Goat Anti-Mouse IgG(H + L) (Proteintech, America) was added and incubated at room temperature for 1 h. The membrane was then washed three times and ECL color developing solution was added for imaging.

### 2.4 Enzyme-linked immunosorbent assay

The recombinant protein (2.5 μg/mL) was used to coat a 96-well microtiter plate overnight at 4°C and then washed three times in 1× ELISA Washing Buffer (Sangon Biotech Co., Ltd., China) for 5 min per wash according to the manufacturer’s instructions. Next, blocking was performed using Blocking Buffer (Sangon Biotech) at 37°C for 1 h, followed by three washes for 5 min per wash. The serum samples and horseradish peroxidase-conjugated secondary antibody (HRP-conjugated Rabbit anti-Goat IgG, HRP-conjugated Goat anti-Bovine IgG, Sangon Biotech) were diluted with General Antibody Dilution Buffer (Sangon Biotech) at a ratio of 1:60 and 1:10,000, respectively. A blank control and positive control were also included. The serum samples were added to each well and incubated at 37°C for 1 h, followed by three washes of 5 min each. The same method is used for secondary antibody. ELISA Color Solution (Sangon Biotech) was added and incubated at 37°C. After 15 min, ELISA Stopping Solution (Sangon Biotech) was added to halt color development. Finally, the optical density was read at 450 nm. The mean value ($\bar{X}$) and standard deviation (SD) of 100 BATV-negative samples were calculated and used to determine the threshold values. Results ≤ $\bar{X}$ + 2 × SD were considered negative, and results ≥ $\bar{X}$+ 3 × SD were considered positive. Results falling between these two parameters were considered suspicious.

The experiment involved the application of the same batch of purified recombinant protein to enzyme-tagged plates, with the selection of 5 positive and 5 negative samples for the assay. Each sample was replicated in triplicate wells. To evaluate interbatch repeatability, three distinct batches of the purified recombinant protein were used on enzyme-tagged plates to test 10 serum samples, including 5 positive and 5 negative. The coefficient of variation was calculated for both within-batch and between-batches to determine the level of repeatability. The BATV antibody-positive serum was subjected to serial dilutions (1:50, 1:100, 1:200, 1:400, 1:800, 1:1600, and 1:3200) to evaluate the sensitivity of the ELISA method. Furthermore, the positive sera of goat pox virus and Brucella were employed to assess the specificity of the ELISA method.

### 2.5 Virus neutralization test

The tested serum samples were inactivated at 56°C for 30 min and double-diluted with FBS-free DMEM. BATV NM/12 suspension was diluted to 100 TCID_50_ with FBS-free DMEM, mixed with equal volumes of serum with different dilutions, and incubated at 37°C for 1 h. The mixtures were added to a 96-well plate containing a monolayer of N2a cells. The same volume of 100 TCID_50_ BATV suspension was added to the virus control well, and the same volume of FBS-free DMEM was added to the negative cell control well. Finally, the plate was incubated at 37°C for 1 h, the supernatant was discarded, PS-DMEM with 2% FBS was added, and the plates were incubated at 37°C in a 5% CO_2_ incubator for 7 days to observe cellular changes.

### 2.6 Statistical analyses and geographical mapping

GraphPad Prism 9 software (GraphPad Software Inc., United States) was used to analyze and plot data. R version 3.5.0 was used to calculate the serum prevalence and 95% confidence interval (CI). R version 3.5.0 was used to draw maps.

### 2.7 Reverse transcription real-time PCR

We referred to the sequence of the BATV coding gene for nonstructural protein NS in the NCBI database (GenBank accession number KJ187040.1) for primer design using Oligo v7.6 software.^3^ The designed primers were BATV-SYF (5′-CTACACCACTGGGC TTAGTTAT-3′; positions 155–177) and BATV-SYR (5′-CGTAACCT CCCATTCACTTCT-3′; positions 234–255), with the amplicon size of 100 bp. The specificity of the designed primers was analyzed using NCBI’s Primer-BLAST.^4^ All primers were synthesized by GENEWIZ (Azenta Life Sciences, United States).

RNA was extracted from the cattle and goat serum samples using a Virus DNA/RNA Extraction Kit (Vazyme) according to the manufacturer’s instructions. The extracted RNA was reverse transcribed using a HiScript II 1st Strand cDNA Synthesis Kit (Vazyme), and the resultant cDNA was used as the qPCR template. Each PCR reaction comprised 10 μL 2× AceQ Universal SYBR qPCR Master Mix (Vazyme), 0.4 μL (10 μmol/L) of each primer, and 2 μL template and was made up to a final reaction volume of 20 μL with sterilized deionized water. The positive control used venom extract RNA and reverse transcription products of BATV (NM/12) with a peak solution-curve tm of 78.2°C. The experiment was repeated three times without adding template cDNA to monitor for contamination. The SYBR Green method is commonly used and can be used for melting curve analysis to ensure only one product is amplified, achieving the experimental goal. The assays were performed on a qTOWER^3^ G Real-Time PCR Thermal Cycler (Jena Bioscience, Germany). The cycling conditions were predenaturation at 95°C for 5 min, followed by 40 cycles of denaturation at 95°C for 10 s, annealing at 58°C for 10 s, and extension at 72°C for 30 s. The reaction melting curve was calculated using the default melting curve of the qTOWER^3^ G Real-Time PCR Thermal Cycler.

## 3 Results

### 3.1 Expression and purification of BATV-Gc_189aa–239aa_ protein

**Figure 1:**
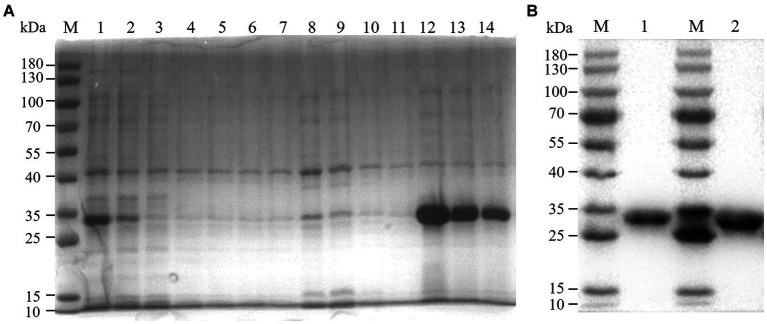
Purification effect of recombinant protein. **(A)** SDS-PAGE. M: 180 kDa marker; 1: bacterial lysate; 2: flow through; 3–6: lysate filtrate; 7–10: washing liquid filtrate; 11: eluent 1; 12: eluent 2; 13: eluent 3; 14: eluent 4. **(B)** western blot. M: 180 kDa marker; 1: eluent 3; 1: eluent 2.

We constructed recombinant plasmid pET-32a-BATV-Gc_189aa–239aa_ and obtained positive clones. Protein expression was induced at 37°C for 10 h with IPTG at a final concentration of 0.6 mM, and the protein was extracted and purified. SDS-PAGE was performed on the bacterial lysate, flow through, lysate filtrate, washing liquid filtrate, and eluent. The electrophoresis results showed bands at 33 kDa (Figure 1A), consistent with the expected size of the recombinant protein. Western blotting showed a specific reaction with the antibody, indicating successful expression of the recombinant protein (Figure 1B), with good antigenicity and specificity. The protein concentration measured using a bicinchoninic acid assay was 0.4076 mg/mL.

### 3.2 Validation of indirect ELISA method

**Figure 2:**
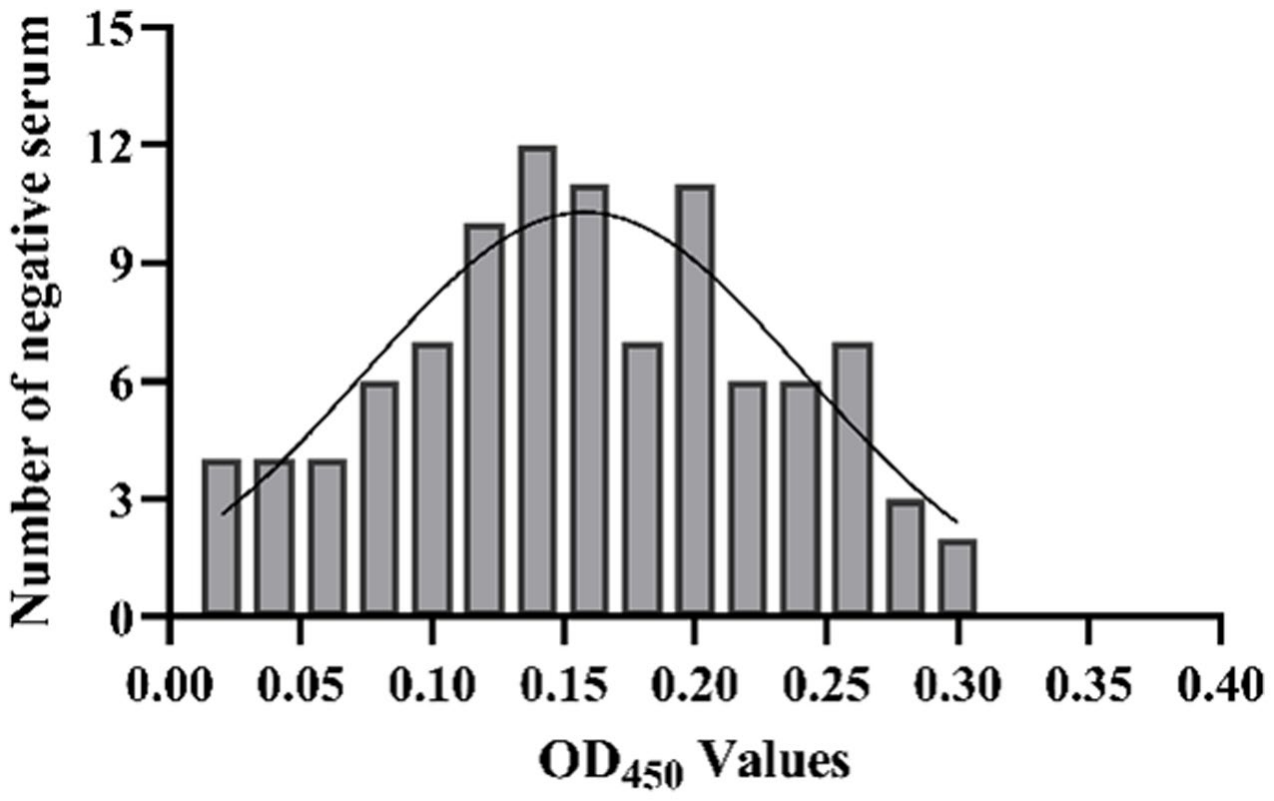
The determination of cut-off value.

**Figure 3:**
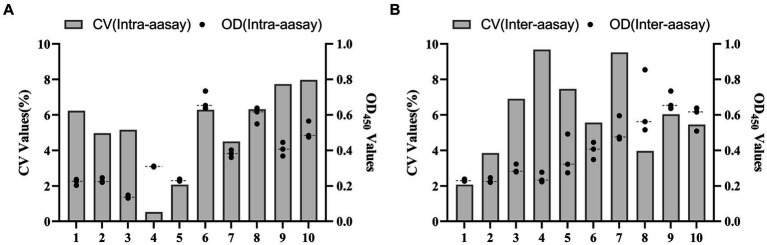
Reproducibility test **(A)** In-lot coefficient. **(B)** Interlot coefficient.

**Figure 4:**
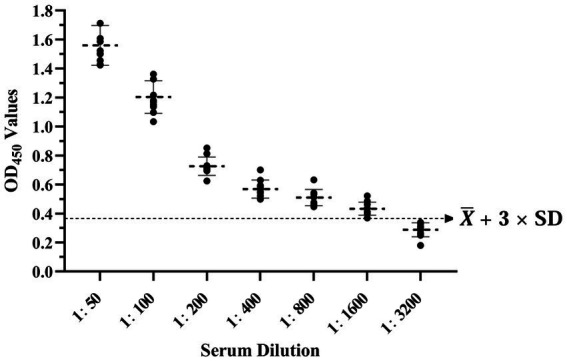
Sensitivity test.

The results showed that the data had a normal distribution based on the Shapiro–Wilk (S-W test:0.174 > 0.05) and Kolmogorov-Smirnova (Sig value: 0.200 > 0.05; Figure 2). The calculated mean ($\bar{X}$) and standard deviation (SD) of OD_450_ are 0.157 and 0.077, and the value of $\bar{X}$+2 × SD and $\bar{X}$+3 × SD are 0.312 and 0.389, respectively. The positive of BATV antibody is indicated by an OD_450_ ≥ 0.389, a negative result by an OD_450_ ≤ 0.312, and a suspicious result by an OD_450_ between 0.312–0.389. The repeatability test showed that the coefficients of variation less than 10% (0.53–7.97%,2.07–9.68%; Figure 3). When the dilution of positive serum was 1:1600, the OD_450_ was greater than 0.389, with high sensitivity (Figure 4). The OD_450_ values for oat pox virus and brucella positive serum were 0.245 and 0.289, respectively, while the OD_450_ value for BATV positive serum was 1.259. This shows that the antigen did not react with goat pox virus or Brucella positive serum, indicating good specificity.

### 3.3 Detection of BATV antibodies in cattle and goat serum by ELISA method

**Figure 5:**
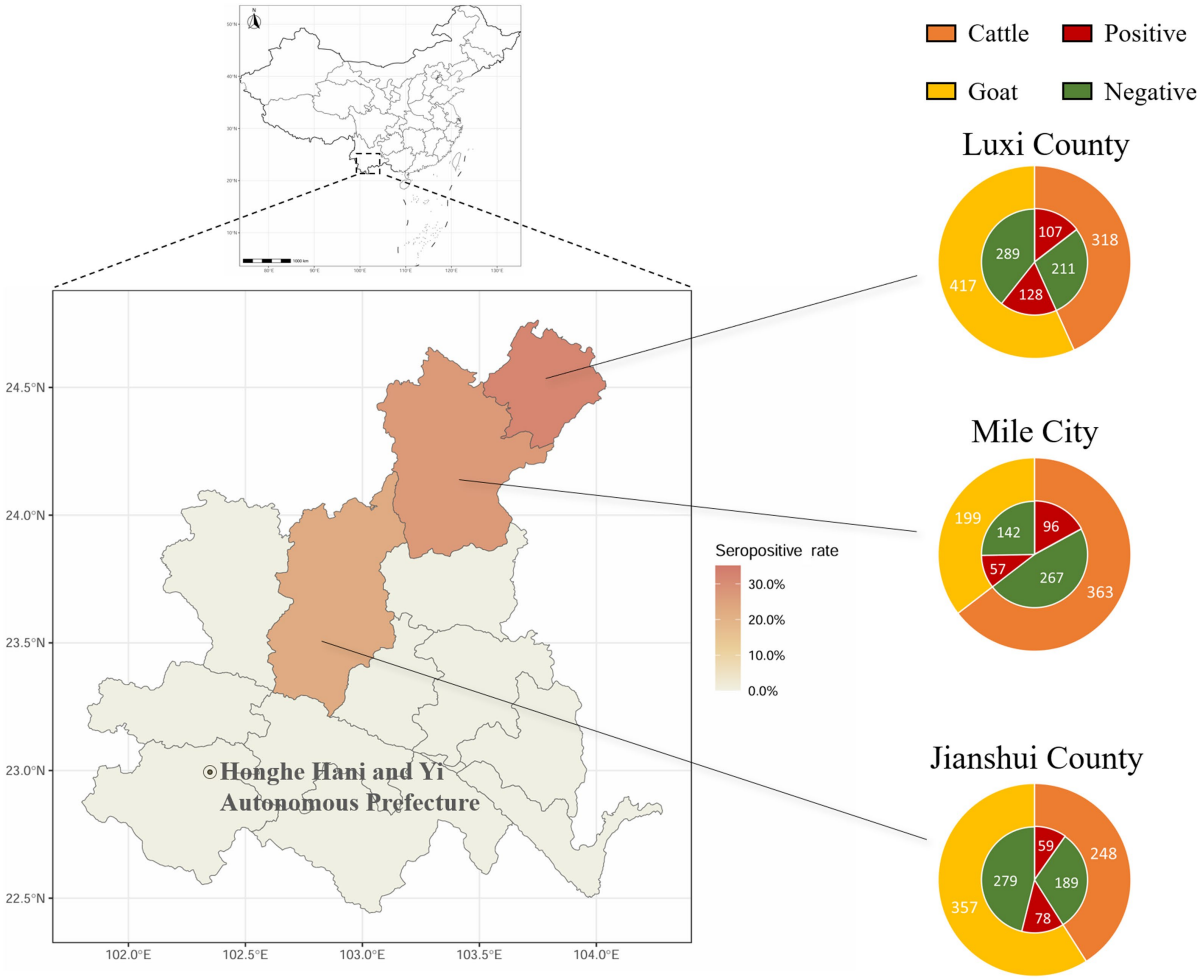
Serological investigation of BATV infection in livestock in Honghe Prefecture, Yunnan Province. Circles represent the number of samples. Different colors in the outer ring represent samples collected from different species. Red in the inner ring represents samples positive for neutralizing antibodies, and green represents samples negative for neutralizing antibodies.

**Figure 6:**
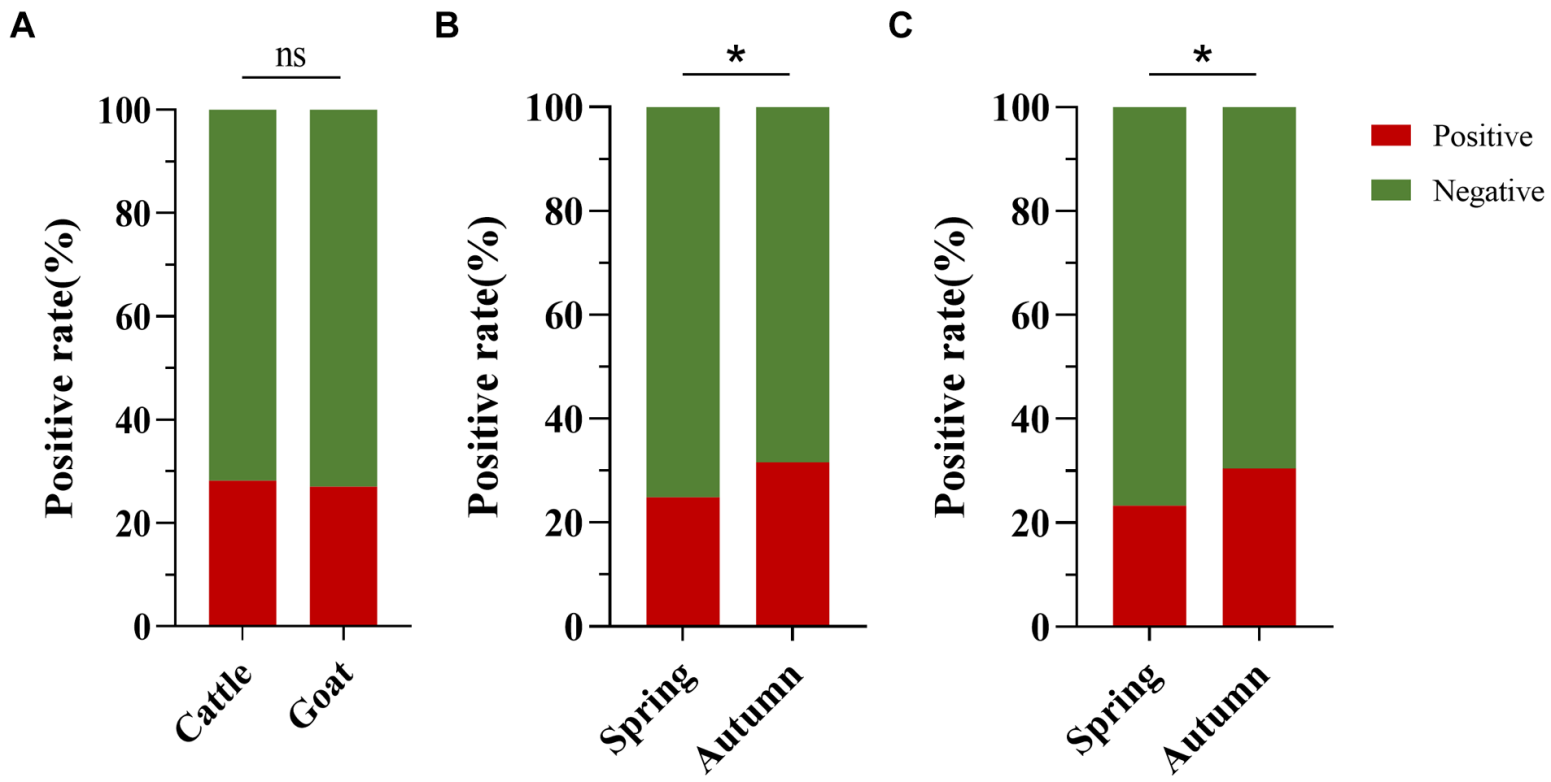
Analysis of BATV antibody detection in cattle and goat serum. **(A)** The positive rate of BATV antibodies in cattle serum was higher than in goats, but the difference was not significant (*p* > 0.05). **(B)** The positive rate of BATV antibodies in cattle serum in autumn was higher than in spring and was statistically significant (*p* < 0.05). **(C)** The positive rate of BATV antibodies in goat serum in autumn was higher than in spring and was statistically significant (*p* < 0.05).

The positive rates of BATV antibodies in cattle and goat serum detected by the established ELISA method were 28.2% (262/929, 95% CI: 25.4–31.2) and 27.0% (263/973, 95% CI: 24.3–29.9), respectively. The positive rates of BATV antibodies in cattle serum samples from Luxi County, Mile City, and Jianshui County were 33.6% (107/318, 95% CI: 28.7–39.0), 26.4% (96/363, 95% CI: 22.2–31.2), and 23.8% (59/248, 95% CI: 18.9–29.5), respectively, and in goat samples, the rates were 30.7% (128/417, 95% CI: 26.5–35.4), 28.6% (57/199, 95% CI: 22.8–35.3), and 21.8% (78/357, 95% CI, 17.9–26.4), respectively (Table 1). The ELISA results indicated that cattle and goats in the three regions could be infected with BATV. Geographically, the positive rate of BATV serum antibody was the highest in Luxi County and the lowest in Jianshui County (Figure 5). The differences in the positive rates of BATV antibodies in cattle and goat samples were not significant (Figure 6A). However, the positive rates in autumn were higher than the positive rates in spring, and the difference was statistically significant (Figures 6B,C). This suggested that BATV prevalence was seasonal.

**Table 1:** The positive rate of BATV in cattle and goat serum in 2021–2022.

Species	Region	Year and season (%)				Total (%)	
		2021		2022			
		Spring	Autumn	Spring	Autumn	Positive rate	95% CI
Cattle	JS	17.4 (8/46)	26.7 (12/45)	22.0 (18/82)	28.0 (21/75)	23.8 (59/248)	18.9–29.5
	ML	19.1 (18/94)	25.5 (24/94)	25.6 (23/90)	36.5 (31/85)	26.4 (96/363)	22.2–31.2
	LX	26.7 (12/45)	32.3 (21/65)	33.3 (39/117)	38.5 (35/91)	33.6 (107/318)	28.7–39.0
Goat	JS	15.1 (14/93)	23.7 (22/93)	21.0 (17/81)	27.8 (25/90)	21.8 (78/357)	17.9–26.4
	ML	19.4 (12/62)	27.7 (13/47)	32.0 (8/25)	36.9 (24/65)	28.6 (57/199)	22.8–35.3
	LX	22.6 (21/93)	27.8 (25/90)	32.5 (37/114)	37.5 (45/120)	30.7 (128/417)	26.5–35.4

*JS, Jianshui County; ML, Mile City; LX, Luxi County.

### 3.4 Neutralization test

A virus neutralization test was carried out on the 525 BATV-positive serum samples (cattle, 262; goats, 263), and the relationship between positive ELISA and neutralization test was shown in Table 2, proving that livestock in Yunnan Province had been infected with BATV.

**Table 2:** Relationship between positive ELISA and neutralization test.

OD_450_	Species	Number of samples	Titers
0.389<OD_450_ ≤ 1	Cattle	227	≤1:8
	Goat	241	
OD_450_>1	Cattle	35	≥1:8
	Goat	22	

### 3.5 Reverse transcription real-time PCR

RT-qPCR was performed on all 929 cattle and 973 goat serum samples. None of the Cq values exceeded the threshold, meaning that the target BATV sequence was not present.

## 4 Discussion

BATV is a newly emerging zoonotic pathogen, and its epidemiological characteristics are closely related to its vector organisms and animal hosts. BATV has been isolated from seals with neurological symptoms in Germany (3), cattle with fever and neurological symptoms (15), and ducks with decreased egg production in China (16). Germany has conducted numerous seroepidemiological investigations on BATV in ruminants. Serological monitoring of cattle, sheep, and goats from 2013 to 2018 revealed that the positive rate of BATV antibodies in cattle serum was as high as 44.7% (13, 18), significantly higher than the rate of 28.2% in our study (*p* < 0.05). Reports on the serological investigation of BATV in cattle and goats in China are scarce. A study on cattle serum from 2012 to 2017 in northern China reported the detection of BATV RNA and antibodies. However, BATV RNA was only detected in the serum of juvenile cattle with neurological symptoms, while the other samples were negative but could induce the body to produce antibodies (15). Currently, there is a lack of research on BATV infection in domestic animals in China, and the absence of a diagnostic kit for BATV antibody poses significant challenges to serological investigation and prevention efforts. In this study, we developed an indirect ELISA method utilizing recombinant BATV-GC_189aa-239aa_ Protein as the coated antigen. Our method demonstrates robust repeatability, high sensitivity, and specificity, making it suitable for high-throughput sample detection. Ours is the first study to investigate BATV in cattle and goat serum in the southwestern border area of China. We tested for the presence of BATV antibodies and BATV RNA using ELISA and RT-qPCR, respectively. The absence of a targeted vaccine for BATV in China suggests that all antibodies are elicited through BATV infection, thereby eliminating the confounding factor of vaccine-induced immunity. The positive rates of BATV antibodies in cattle and goat serum from the same region were similar, and there were no significant differences. All three regions in our study are located in the subtropical monsoon area, characterized by rainy summers and a high density of mosquitoes. The Nanpan River passes through Luxi County and Mile City, and the river and the surrounding wetlands are excellent breeding grounds for mosquitoes. Additionally, some areas of Yunnan Province are prone to floods and mudslides. Luxi County experienced floods in August 2021 and September 2022, and floods facilitate the mass breeding of mosquitoes, increasing the chances of animals being bitten and infected with vector-borne viruses. This may contribute to the high positive rate of BATV antibodies in Luxi County and Mile City. Our study confirmed the presence of BATV antibodies in the serum of cattle and goats in the region, and there was no significant difference in the positive rate of BATV serum antibodies between the two groups. In positive samples, the OD_450_ value measured by ELISA may be positively correlated with serum titer. Notably, the positive rate of BATV antibodies in cattle serum in Yunnan Province in our survey was significantly higher than previous survey results in northern China, including Inner Mongolia Autonomous Region, Heilongjiang Province, and Jilin Province. This may be because the region borders Vietnam, where mosquitoes are extremely active, and frequent cattle and goat trade across the border increases the chances of cattle and goats being bitten and infected with vector-borne viruses. Our findings prove that BATV has appeared and spread among ruminants in southwestern China. However, BATV RNA was not detected in our survey, which is consistent with the results of previous studies in Germany (13, 19), where BATV RNA was also not detected in cattle and goat serum samples. Similarly, Akabane virus, a member of the *Orthobunyavirus* genus, was detected in cattle brain samples using a one-step multiplex reverse-transcriptase-qPCR method (1/123) but not in 100 bovine serum samples or 100 goat serum samples (20). This may be because the virus has a short lifespan in the animal body, and the animals did not have viremia at the time of sampling. Additionally, recent studies have confirmed that besides livestock, birds, such as the gray partridge (*Perdix perdix*), carrion crow (*Corvus corone*), and gray goose (*Anser anser*), are susceptible to BATV in Europe (21). This suggests that birds serve as important hosts for BATV and spread the virus to other regions through migration. Therefore, the scope of host monitoring should be expanded, and targeted monitoring and research on migratory and wild birds should be conducted to determine their role in BATV spread.

## 5 Conclusion

Our study confirmed BATV infection of cattle and goats in the border area between China and Vietnam from 2021 to 2022, and the positive rate of BATV antibodies showed seasonal and regional distribution patterns. Luxi County had the highest positive rate, and the positive rate in autumn was higher than in spring. These findings may closely related to the number of mosquitoes and the environment in each region. Therefore, mosquito control should be strengthened, and the scope of BATV host monitoring should be expanded.

## Data availability statement

The original contributions presented in the study are included in the article/Supplementary material, further inquiries can be directed to the corresponding author/s.

## Ethics statement

The animal studies were approved by the Institutional Animal Care and Use Ethics Committee (IACUC) of the Chinese Academy of Military Medical Science, Changchun, China (10ZDGG007). The studies were conducted in accordance with the local legislation and institutional requirements. Written informed consent was obtained from the owners for the participation of their animals in this study.

## Author contributions

ZL: Formal analysis, Investigation, Writing – original draft. XY: Formal analysis, Investigation, Writing – original draft. GF: Software, Validation, Writing – review & editing. LL: Validation, Writing – review & editing. XS: Writing – review & editing, Resources. HuiL: Resources, Writing – review & editing. NJ: Resources, Writing – review & editing, Supervision. HaoL: Supervision, Writing – review & editing, Funding acquisition. WS: Supervision, Writing – review & editing.

## Footnotes

^1^NCBI, https://www.ncbi.nlm.nih.gov/

^2^NCBI, https://www.iedb.org/

^3^ https://www.oligo.net/downloads.html

^4^ https://www.ncbi.nlm.nih.gov/tools/primer-blast/
