# Supplementary material for: Molecular and serological investigations of Batai virus in cattle and goats in the border area of Yunnan, China (2021–2022)
Source: Front Vet Sci. 2024 Jul 31;11:1433699. doi: 10.3389/fvets.2024.1433699 (PMC11322338; doi:10.3389/fvets.2024.1433699)
Supplement: Supplementary file 1 [file Data_Sheet_1.pdf]

Supplementary Material

Molecular and serological investigations of Batai Virus in Cattle and Goats in the Border Area of Yunnan, China (2021–2022)

Zishuo Lu[1†], Xingxiu Yan[1†], Guiying Fan[1], Lixia Li[1], Xiutao Sun[2], Huijun Lu[3], Ningyi Jin[3], Hao Liu[1*], Wenchao Sun[4*]

*** Correspondence:**
Hao Liu
liuhao_lh@hotmail.com

Wen-Chao Sun
Email:sunwenchao_131@126.com

1 Supplementary Figures

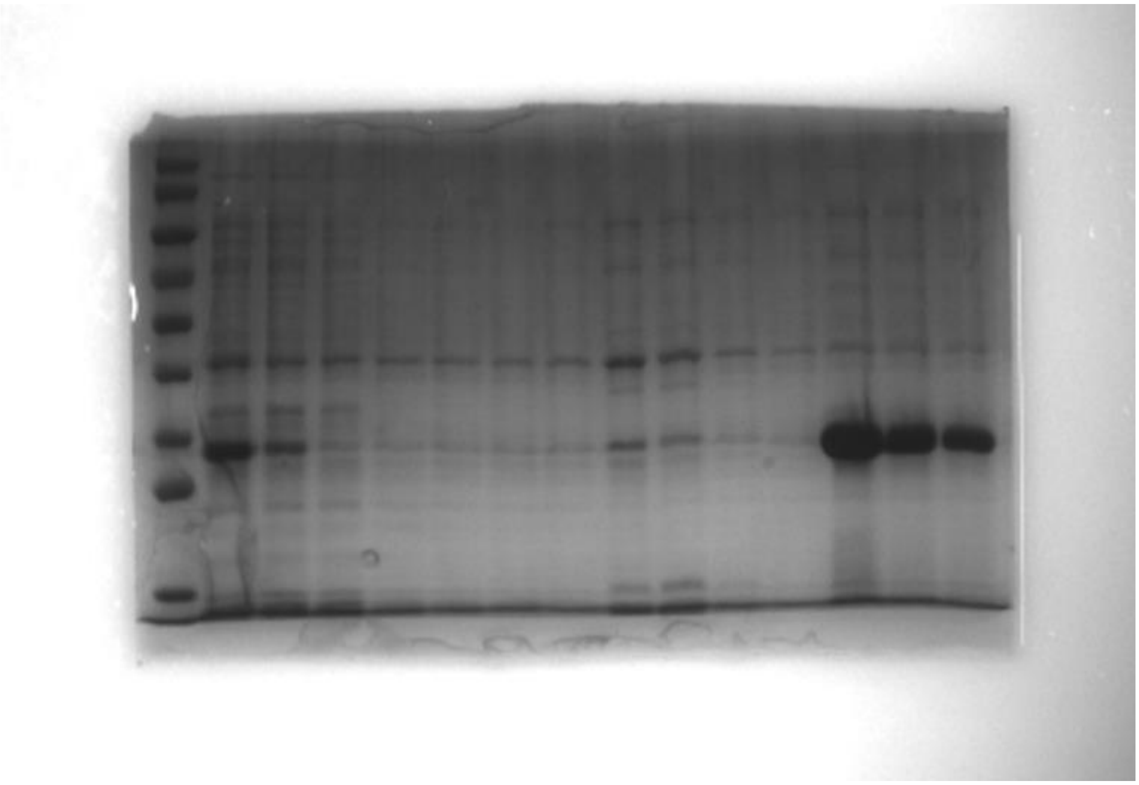

Figure S1. The original image files of western blot in Figure 1 (A)

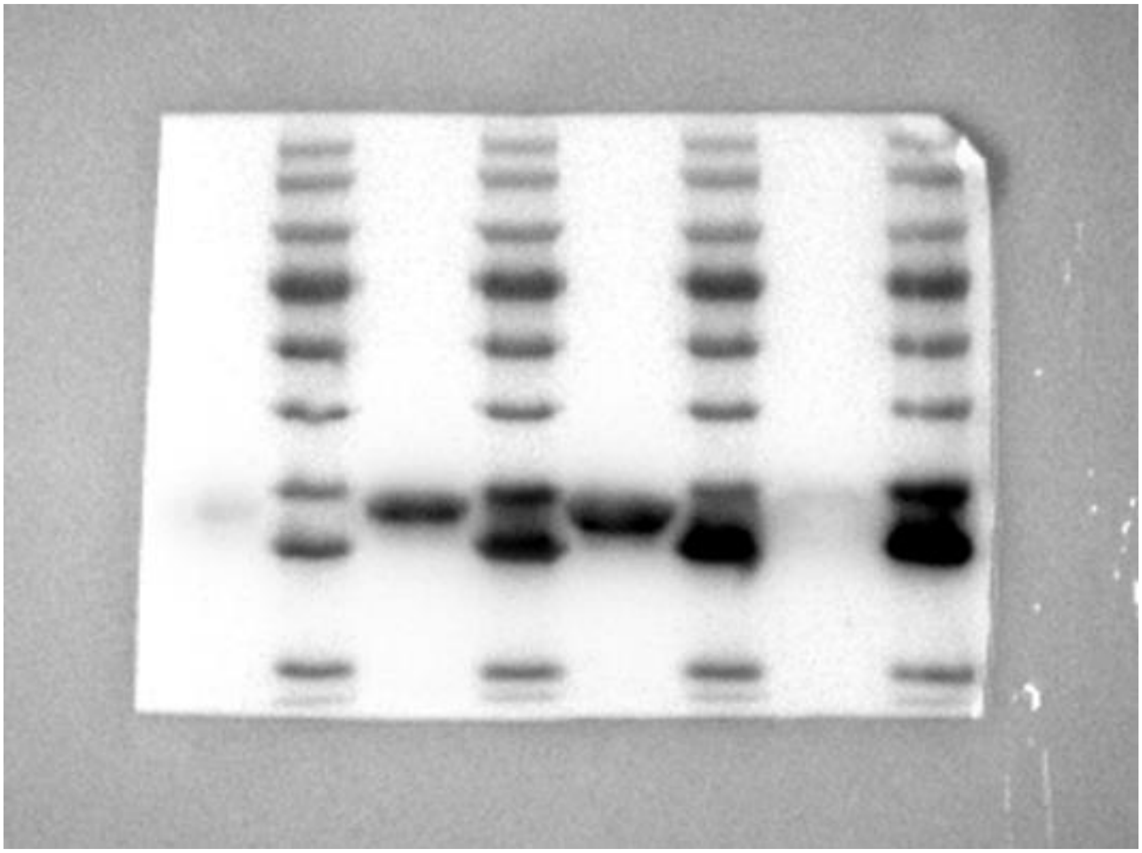

Figure S2. The original image files of western blot in Figure 1 (B)

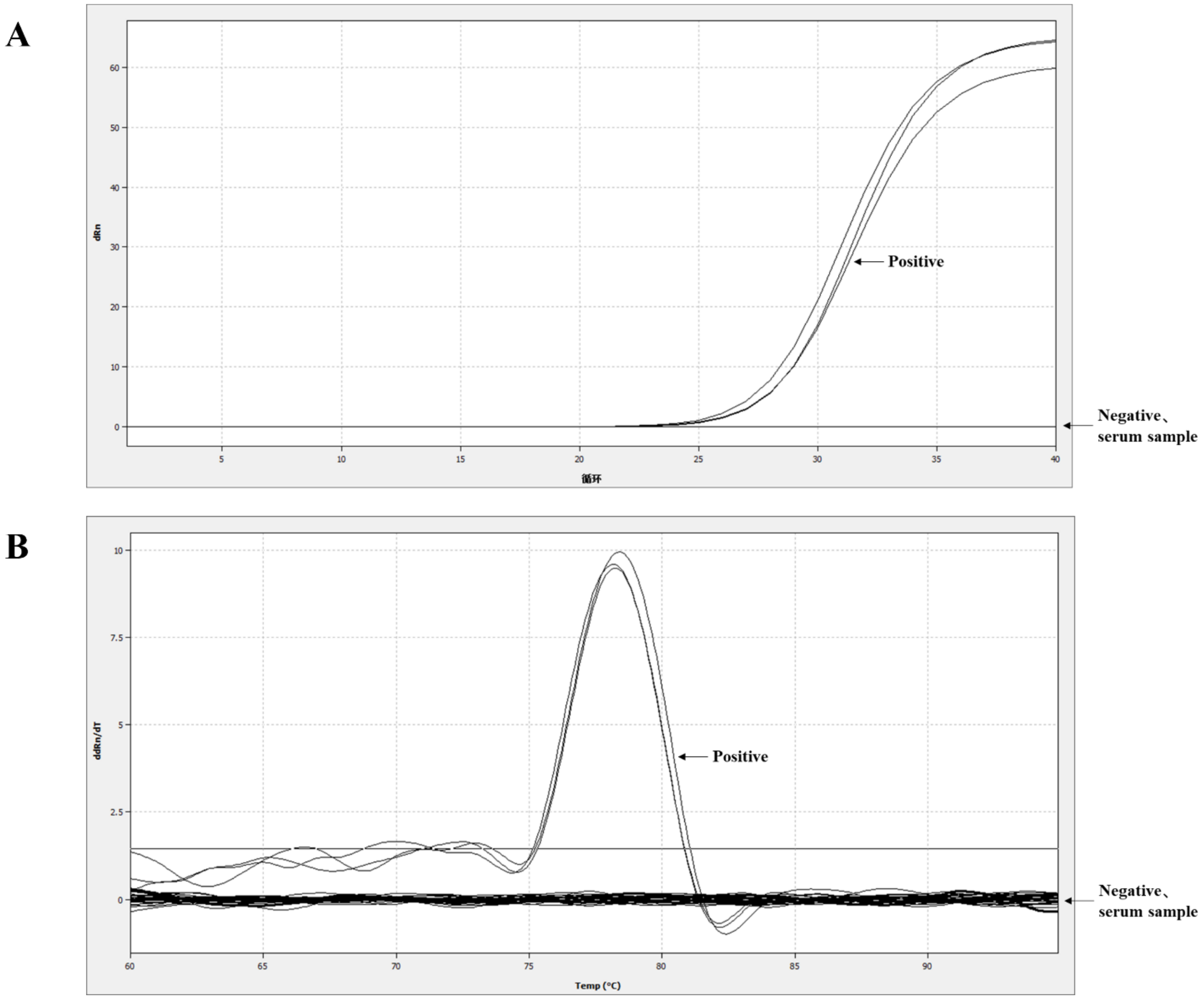

Figure S3. RT-qPCR results. (A): Amplification map; (B): Dissolution curve.
